# Changes in Choroidal Thickness and Retinal Activity with a Myopia Control Contact Lens

**DOI:** 10.3390/jcm12113618

**Published:** 2023-05-23

**Authors:** Ana Amorim-de-Sousa, Jaume Pauné, Sara Silva-Leite, Paulo Fernandes, José Manuel Gozález-Méijome, António Queirós

**Affiliations:** 1 Clinical and Experimental Optometry Research Lab (CEORLab), School of Science, University of Minho, 4710-057 Braga, Portugal; ana.amorim.sousa@gmail.com (A.A.-d.-S.); pfernandes@fisica.uminho.pt (P.F.);; 2 Teknon Medical Center, 08022 Barcelona, Spain; 3 Faculty of Optics and Optometry Polytechnic, University of Catalonia, 08222 Terrassa, Spain; 4 Physics Center of Minho and Porto Universities, CF-UM-UP, 4710-057 Braga, Portugal

**Keywords:** myopia control, contact lens, choroidal thickness, ERG, retinal activity

## Abstract

Purpose: The axial elongation in myopia is associated with some structural and functional retinal changes. The purpose of this study was to investigate the effect of a contact lens (CL) intended for myopia control on the choroidal thickness (ChT) and the retinal electrical response. Methods: Ten myopic eyes (10 subjects, 18–35 years of age) with spherical equivalents from −0.75 to −6.00 diopters (D) were enrolled. The ChT at different eccentricities (3 mm temporal, 1.5 mm temporal, sub-foveal ChT, 1.5 mm nasal, and 3 mm nasal), the photopic 3.0 b-wave of ffERG and the PERG were recorded and compared with two material-matched contact lenses following 30 min of wear: a single-vision CL (SV) and a radial power gradient CL with +1.50 D addition (PG). Results: Compared with the SV, the PG increased the ChT at all eccentricities, with statistically significant differences at 3.0 mm temporal (10.30 ± 11.51 µm, *p* = 0.020), in sub-foveal ChT (17.00 ± 20.01 µm, *p* = 0.025), and at 1.5 mm nasal (10.70 ± 14.50 µm, *p* = 0.044). The PG decreased significantly the SV amplitude of the ffERG photopic b-wave (11.80 (30.55) µV, *p* = 0.047), N35-P50 (0.90 (0.96) µV, *p* = 0.017), and P50-N95 (0.46 (2.50) µV, *p* = 0.047). The amplitude of the a-wave was negatively correlated with the ChT at 3.0T (r = −0.606, *p* = 0.038) and 1.5T (r = −0.748, *p* = 0.013), and the amplitude of the b-wave showed a negative correlation with the ChT at 1.5T (r = −0.693, *p* = 0.026). Conclusions: The PG increased the ChT in a similar magnitude observed in previous studies. These CLs attenuated the amplitude of the retinal response, possibly due to the combined effect of the induced peripheral defocus high-order aberrations impacting the central retinal image. The decrease in the response of bipolar and ganglion cells suggests a potential retrograde feedback signaling effect from the inner to outer retinal layers observed in previous studies.

## 1. Introduction

The possible structural and functional consequences of high myopia due to axial elongation are the major concerns of the scientific and clinical communities, as its incidence and progression are increasing [1]. Early myopia onset frequently leads to high myopia levels before fourteen years of age, and the disproportional increasing rate of myopia progression [2] increases the risk of high myopia. In some countries, myopia is considered a public health issue [3], leading to an increasing interest in developing treatments that decrease the rate of eye elongation to reduce the likelihood of future severe visual loss.

Aside from retinal nutrition, the choroid might play an active key role in emmetropization based on eye growth mechanisms [4], which makes it a potential signaling factor to control the onset and progression of myopia [5,6,7]. However, the exact mechanism of action is not yet understood. Choroidal thinning is one of the structural features of myopia, with a tendency to correlate significantly with the myopia magnitude: the higher the myopia levels, the more the eye and retina are stretched, and consequently, the thinner the choroid [8,9,10,11].

Several studies showed that the choroidal thickness (ChT) changes according to the defocus imposed on the retina (positive or negative defocus). These studies are concordant in most respects and show an increase in ChT when exposed for a period of time to positive (myopic) defocus, while imposing a negative (hypermetropic) defocus produces a ChT decrease [12,13,14,15,16,17]. Some of the methods used for myopia control also change the ChT, such as atropine [18], orthokeratology [19,20,21,22], and multifocal contact lenses [23].

There are some differences in the retinal function between myopic and non-myopic eyes. Several studies reported a decrease in amplitude or a slight delay in peak response time in myopic eyes [24,25,26,27]. These findings were more evident for higher refractive errors [24,25,28], with amplitudes decreasing between 5 and 10% for each millimeter of axial elongation [28,29]. These changes were observed with techniques that assess the contribution of bipolar cells, although ganglion cells are more sensitive to changes in contrast [30]. Additional significant decreases in retinal function have been observed in progressing myopes compared to emmetropes or non-progressing myopes across the entire retina [31]. While there is a shared agreement that myopia and axial elongation affect retinal functionality, there is no consensus on which areas (central versus peripheral retina) have the greatest influence and where the response is most affected.

Myopia control devices show a 30–69% efficacy in retaining the rate of eye growth compared to the control groups [32,33]. Several hypotheses have been raised in an attempt to understand the exact cascade of mechanisms for defocus detection that signals the eye to grow slower and, consequently, reduce myopia progression through myopia management devices. Some studies suggest that choroid may have an active role in the emmetropization process due to changes in ChT as a consequence of imposed defocus [13,14,15,34]. It is also suggested that the shift in the peripheral retinal defocus induced by myopia control devices changes the signaling through the retinal activity to slow the rate of eye growth. However, the exact mechanism of action of both choroidal and cellular activity processes is not yet well elucidated.

The present pilot study aims to evaluate the possible structural and functional changes in the retina of young adults with stable myopia through the measurement of the ChT at different symmetric retinal eccentricities and recording the electrophysiological response of the inner and outer retina when using a contact lens with the potential for myopia control. It was hypothesized that the design of these types of lenses by itself likely influences the cellular response due to the induced defocus, even though no relationship between the structural and functional changes may be found.

## 2. Methods

### 2.1. Study Design and Recruitment

In this cross-sectional pilot study, the changes in ChT and the retinal electrical activity were evaluated after 30 min of use of a CL with the potential for myopia control and a single-vision CL. The study was conducted at CEORLab (University of Minho, Portugal) in collaboration with Precilens (Creteil, France), donating the lenses. The protocol complied with the guidelines of the Helsinki Declaration and was approved by the Ethics Committee for Research in Life and Health Sciences (CEICVS 038/2019, 26 July 2019) of the Ethics Council of the University of Minho (CEUMinho). All subjects had access to detailed information on the study’s purpose and procedures and were asked to give and sign an informed consent.

Inclusion criteria were as follows: 18 to 35 years of age, spherical equivalent between −0.75 D and −6.00 D, astigmatism less than 1.00 D, best spectacle-corrected visual acuity of 0.1 logMAR or better, transparent ocular media, no ocular or systemic health conditions, and no previous history of ophthalmic surgical intervention or myopia control treatment. Subjects exceeding a myopia increase superior to 0.50 D over the last year were excluded [35].

### 2.2. Protocol

Two visits were scheduled. The 1st visit was required to verify the eligibility of the subjects for the study. An ophthalmic examination of both eyes was carried out, including refraction, ocular media integrity and fundus evaluation, high-contrast corrected visual acuity, and a registry of the clinical history of ocular and systemic health.

The 2nd visit was scheduled within a maximum of 7 days. The high and low contrast visual acuity (HCVA and LCVA, respectively), the choroidal thickness (ChT), and visual electrophysiology measurements using an electroretinogram (ERG) were recorded after 30 min of a single-vision contact lens (SV) use. The former measurements were repeated with a CL designed for myopia control purposes, with a radial power gradient design. After the removal of the SV, there was a washout period of 15 min. All measurements were performed by a single investigator.

### 2.3. Material and Procedures

Two optical designs of CLs of the same material (Benz G3X p-GMA/HEMA—Hioxifilcon B, GM advance 49% water content, 15 Dk—35 °C Fatt Units, Benz, Sarasota, United States) were used: a spherical SV and a radial power gradient contact lens (PG) produced for myopia management purposes. The PG has a single progressive optical zone up to +1.50 D of addition at 2 mm semi-chord from the center, achieving +6.50 D at the edge of the optic zone (4 mm semi-chord) [36]. Both lenses had a diameter of 14.5 mm and were available in three different radii of curvature (8.10 mm, 8.40 mm, and 8.70). For each subject, the CLs radius of curvature was chosen according to the individual mean corneal radius of curvature plus 1.0 mm, rounded up to the nearest radius of curvature available. Before any measurements, the subjects had to have been wearing the lenses for 30 min.

The high contrast (100% contrast) and low contrast (10% contrast) were measured (logMAR units) both with the SV and the PG using the Early Treatment Diabetic Retinopathy Study chart at 4 m distance from the observer. 

**Figure 1:**
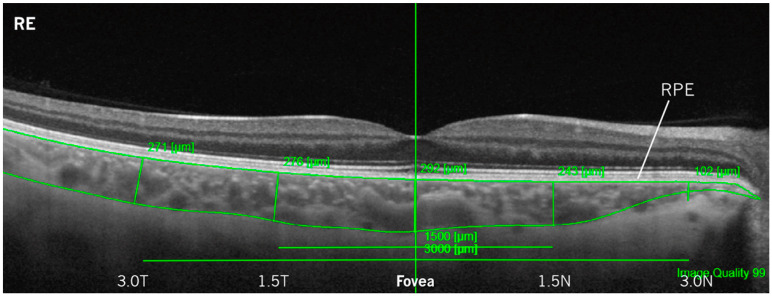
Example of an OCT scan from DRI-OCT TritonTM with layer segmentation (from the retinal pigment epithelium—RPE, upper limit—to the outermost limit with visible vascularization—lower limit) for choroidal thickness measurements at different eccentricities (temporal and nasal sides—right and left, respectively) of a right eye (RE).

The ChT measurements were assessed through optical coherence tomography (OCT) acquisition with the DRI-OCT TritonTM (Topcon, Japan) with both SV and PG, using a linear scan of 12 mm length with fixation position on the macula (1024 resolution, overlaps 128 data reads). The OCT acquisitions were performed 30 min after the use of each contact lens design, following visual acuities assessment, and the image quality should be 90% or better. The ChT—distance from the retinal pigment epithelium to the outermost limit with visible vascularization—was obtained using the manual measurement tool of the software at five retinal eccentricity points: 3 mm temporal (3.0 T), 1.5 mm temporal (1.5 T), at the fovea (sub-foveal ChT), 1.5 mm nasal (1.5 N), and 3 mm nasal (3.0 N), as shown in the example from Figure 1.

The electrical response of the retina was assessed with the RETI-port/scan21 (Roland Consult, Wiesbaden, Germany) using two ERG tests—the photopic light-adapted 3.0 full-field ERG (photopic b-wave) and the pattern ERG—following the ISCEV guidelines [37,38]. The first allows for assessing the activity of cones and mostly ON-type bipolar cells, while the pattern ERG allows for assessing the global response of ganglion cells.

Pupils were fully dilated with two drops of 1% Phenylephrine (Davinefrina, DÁVI II—Farmacêutica S.A, Portugal) 25–30 min before ERG recordings. The skin was cleansed with an abrasive gel prior to electrode placement (gold-cup reference, ground electrodes, and an active DTL-plus electrode). All measurements were performed binocularly and with signal impedance below 10 kOhm.

**Figure 2:**
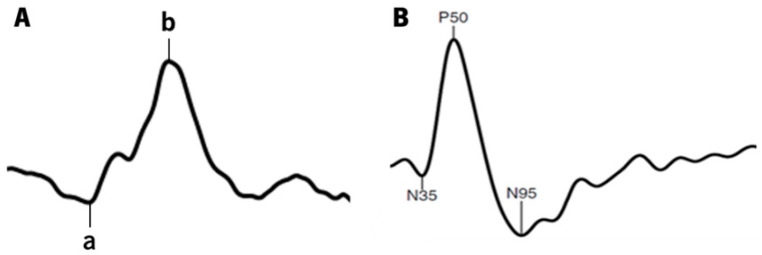
Typical waveforms of the ffERG photopic 3.0 response (**A**) and pattern ERG response (**B**), with the correspondent wave-peaks (a-wave and b-wave in ffERG photopic 3.0, and N35, P50 and N95 in pattern ERG).

In the photopic b-wave, a sequence of 5 single white light flashes (3.0 cd.s/m^2^) is generated in a Ganzfeld dome against a white background (30 cd/m^2^) at a rate of 0.625 Hz and a recording bandpass filter of 1–300 Hz. Subjects were asked to position properly on the chin and forehead rests and maintain their fixation on a central light inside the dome. The resulting response is a waveform similar to Figure 2A.

In the transient pattern ERG response, the retinal activity is generated by a checkerboard pattern stimulus with reversible black and white squares (1.53 rev/s) presented on an LCD monitor (ProLite B1980SD, Iiyama, Tokyo, Japan) with a frame rate of 60 Hz. The test is performed at a viewing distance of 1 m, covering a field size of 15°. The squares have a size of 0.8° and an average illuminance of 152.65 ± 0.64 lux (Illuminance meter T-10A, Konica Minolta, Osaka, Japan). The mean luminance for the black squares was 1.47 ± 0.06 cd/m^2^, and for the white squares, it was 220.32 ± 1.23 cd/m^2^ (Luminance meter LS-150, Konica Minolta, Osaka, Japan). The signal was amplified and passed through a bandpass filter of 5–50 Hz, resulting in an average curve with a sweep length of 180 ms (sample freq. 2.84 Hz), similar to the one depicted in Figure 2B. Subjects are required and reminded to maintain fixation on a red cross in the center of the checkerboard throughout the recording.

### 2.4. Statistics Analysis

The sample size was calculated (GPower 3.1 software) considering a two-sided effect with a mean difference of 15 µm (standard deviation, SD = 10 µm) in ChT16 and a mean difference of 1.5 µV (SD = 1 µV) in P50 pattern ERG amplitude [39] between the SV and PG conditions. The p-value was set at 0.05 and with a statistical power of at least 0.80. Only one eye (randomly chosen) per subject was analyzed since it was shown that the two eyes of each subject were not independent (*r* > 0.95, *p* < 0.001) for any of the variables.

Statistical analysis was carried out using IBM SPSS Statistics v28.0 (IBM Inc., Armonk, NY, USA) software. The normality of the data distribution was checked with the Shapiro–Wilk test. Parametric tests (*t*-test, Pearson correlation) were used for variables with a normal distribution (shown as mean ± standard deviation—mean ± SD), while non-normal variables were analyzed with non-parametric tests (Wilcoxon test and Spearman correlation; values shown as the median and interquartile range—median (IQR)).

## 3. Results

### 3.1. Sample Characteristics and Visual Acuity

Ten individuals (29.2 ± 6.7 years) participated in the study (five right eyes and five left eyes). The mean vectorial components of refraction were M = −3.30 ± 1.35 D, J0 = −0.12 ± 0.20 D, and J45 = −0.01 ± 0.03 D. The high contrast (HCVA) and low contrast (LCVA) visual acuities of the two CLs were very similar (HCVA_SV_ = −0.03 ± 0.08 logMAR, HCVA_PG_ = −0.06 ± 0.04 logMAR, LCVA_SV_ = 0.19 ± 0.10 logMAR, and LCVA_PG_ = 0.24 ± 0.09 logMAR), with no statistically significant differences between them (*p* > 0.050).

### 3.2. Choroidal Thickness

**Figure 3:**
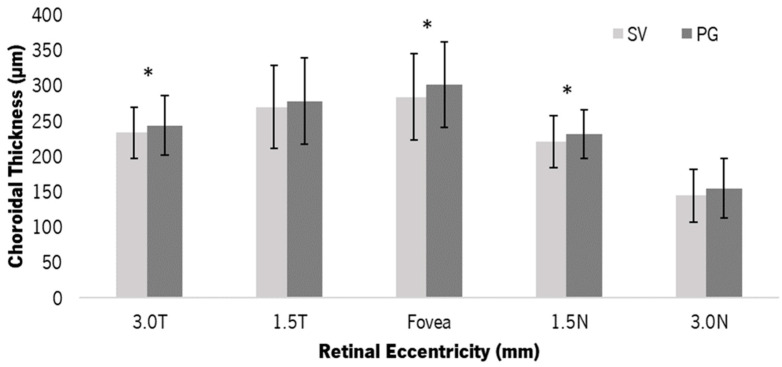
Average (and SD bars) of the ChT values with the SV and PG for the five eccentric choroidal zones. The ChT with the PG (dark grey) was thicker than the SV (light grey) after 30 min of wear. * Statistically significant differences between conditions (*t*-test, *p*-value ≤ 0.050).

Figure 3 shows the mean ChT at each retinal point with the SV and the PG. After 30 min of lens wear, the PG showed thicker choroids compared to the SV. The differences in ChT were statistically significant at 3.0 T (mean difference of 10.30 ± 11.51 µm, 95% IC (2.07 to 18.53), *p* = 0.020), in sub-foveal ChT (mean difference of 17.00 ± 10.01 µm, 95% IC (2.69 to 31.32), *p* = 0.025) and at 1.5 N (mean difference of 10.70 ± 14.50 µm, 95% IC (1.33 to 21.07), *p* = 0.044). At 3.0 N, the difference between lenses was close to statistical significance (mean difference of 10.50 ± 15.37 µm, 95% IC (1.50 to 21.50), *p* = 0.059). There were no statistically significant differences between temporal and nasal points of the same eccentricity.

### 3.3. Electrophysiology—ffERG—Photopic 3.0 Response

**Figure 4:**
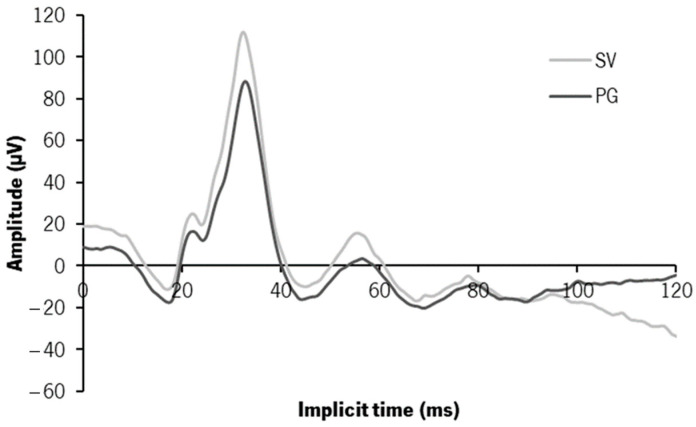
Median curves of the photopic 3.0 ffERG response recorded 30 min after wearing the single-vision CLs (SV in light grey) and the power gradient CLs (PG in dark grey).

Figure 4 shows curves of the photopic 3.0 response with the SV (light grey) and the PG (dark grey). The median (IQR) values of the implicit time (in milliseconds—ms) and amplitude (in microvolts—µV) of the two main peaks (a- and b-waves) and the median differences between CLs are in Table 1.

There was a negative shift of the response curve with PG relative to SV from the initial time frame (0 ms) without any significant changes in the time-to-peak. The mean voltage values at the a- and b- peaks with the PG were 6.49 ± 13.44 µV and 21.88 ± 21.70 µV, respectively, more negative, with statistically significant differences for the b-wave (Z = −2.599, *p* = 0.009). A statistically significant delay and decrease in the amplitude of the b-wave were observed with the PG (Table 1). The delay was not considered to be clinically significant since this was a very low difference within the range of intrasubject variation. Regarding the amplitude, the statistical analysis showed a statistically significant decrease of 2–17% in b-wave amplitude (Table 1).

### 3.4. Electrophysiology—Pattern ERG Response

**Figure 5:**
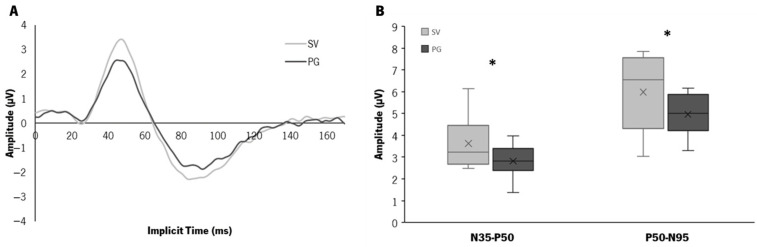
(**A**) Median curves of pattern ERG response with SV (light grey) and PG (dark grey). (**B**) Distribution of the amplitude values of the pattern ERG response (N35-P50 and P50-N95) obtained with the two types of lenses (SV in light grey, PG in dark grey). (*) Statistically significant differences, *p* ≤ 0.050, Wilcoxon test.

Figure 5A shows the median pattern ERG response curves obtained in the two conditions. The implicit median time of the three peaks (N35, P50, and N95) of the two contact lenses were very similar, without statistically significant differences (N35_SV_ = 24.64 (3.08) ms, N35_PG_ = 24.64 (2.64) ms, P50_SV_ = 47.00 (3.61) ms, P50_PG_ = 44.88 (5.37) ms, N95_SV_ = 86.77 (6.25) ms, N95_PG_ = 90.82 (12.50) ms).

The median (IQR) amplitudes with the SV were N35-P50_SV_ = 3.23 (1.79) µV and P50-N95_SV_ = 6.55 (3.26) µV, and with the PG were N35-P50_PG_ = 2.83 (1.00) µV and P50-N95_PG_ = 5.00 (1.65) µV (Figure 5B). The PG showed a significantly lower N35-P50 amplitude of 0.90 (0.96) µV (*p* = 0.017) and a lower P50-N95 amplitude of 0.46 (2.50) µV (*p* = 0.047) compared to SV. Although the magnitude of the differences was low, there appears to be a shorter range of amplitudes with the PG compared to the SV (Figure 5B). These changes represent between 21% and 45% of the amplitudes obtained with the SV.

### 3.5. Choroidal Thickness and Electroretinogram

**Figure 6:**
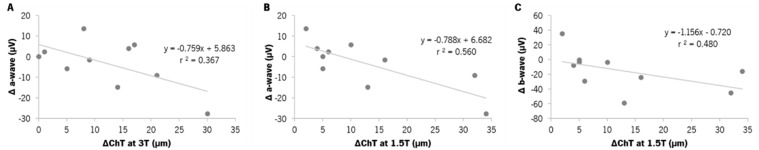
Statistically significant Spearman’s correlation graphs of the differences between PG and SV in choroidal thickness with the difference in ffERG a- and b-wave amplitudes. (**A**,**B**) shows the relationship between the changes in a-wave amplitude and the increase in ChT at 3.0 mm and 1.5 mm temporal side, respectively. (**C**) shows the relationship between the changes in b-wave amplitude with the increase in ChT at 1.5 mm on the temporal side.

After the comparison between CLs in ChT and ERG measurements, it was investigated whether the differences in ChT could be related to the changes in the amplitude parameters from ERG responses. Spearman’s correlation showed statistically significant negative and moderate correlations between the changes in amplitudes of a- and b-wave with the changes in the temporal ChT (Figure 6). The amplitude of the a-wave decreased with choroidal thickening at 3.0T (r = −0.606, r^2^ = 0.367, *p* = 0.038, Figure 6A) and 1.5T (r = −0.748, r^2^ = 0.560, *p* = 0.013, Figure 6B), as the amplitude of the b-wave decreased with the thickening of the choroid at 1.5T (r = −0.693, r^2^ = 480, *p* = 0.026, Figure 6C). However, these correlations showed considerable dispersion of the data.

## 4. Discussion

The present study evaluated the impact of a radial power gradient contact lens (PG) on the choroidal thickness (ChT) and retinal activity of young myopic subjects after 30 min of lens wear. The outcomes were compared with a single-vision contact lens (SV). The visual performance in terms of visual acuity at high and low contrast with the two lenses was very similar. From the authors’ knowledge, this is the first study to analyze the impact of using a soft CL with the potential for myopia control on the measurement of the ChT and the electrophysiological response of the retina in the same cohort of subjects.

### 4.1. Structural Changes—Choroidal Thickness

In the present study, the PG induced an increase in ChT at all retinal eccentricity points after 30 min of wear, with significant changes in some points. The average increment in sub-foveal ChT of 17.00 µm with the PG is within the range previously observed in other human studies with different types of defocus imposition. Read et al. [15] were the first to observe this behavior in humans after inducing defocus of 3.00 D (myopic and hyperopic) for 60 min. They observed a choroidal thickening of about 12 µm with the positive defocus and a decrease of about 3 µm with the negative defocus. Later, Chiang et al. [16] observed a choroidal thickening of 15–20 µm after 60 min of imposing +2.00 D of defocus and a choroidal thinning of the same magnitude with −2.00 D defocus. Other studies involving children under orthokeratology treatment have also reported an increase in sub-foveal ChT of at least 20 µm compared to the control group [19,20].

The nasal choroid is naturally thinner than the temporal choroid [40]. The increase in ChT with the PG occurred in both nasal and temporal eccentric points (1.5 and 3.0 mm) but at a lower magnitude in the nasal points. Chen and colleagues [20] observed a greater thickening of the temporal parafoveal choroid compared to the nasal choroid after 3 weeks of orthokeratology treatment. They suggested that the thicker areas of the choroid result in a greater ability to increase upon a defocus stimulus. More recently, Hoseini-Yazdi and colleagues [41] observed that, after 60 min of imposing hemifield myopic blur, only the ChT in the hemifield exposed to the defocus increased, suggesting that the response mechanism to defocus is regionally localized. However, in the present study, the increment was very similar for different eccentric points and in the nasal and temporal choroid, and a higher thickening of the sub-foveal ChT. This might be related to the optical profile of the lens, as well as to the methodology for the ChT measurement, other than the sample number, age, and defocus period. 

The power gradient defocuses profile differs from that of orthokeratology CLs. Although both are expected to increase the comatic and spherical-like aberrations in the overall image, in the PG, the increase occurs at a constant level across the sample as the design is the same irrespective of the treatment, while in orthokeratology, the defocus levels increase with myopic prescription [42,43]. While in the present study, the measurements of ChT were performed manually by the same user through the equipment’s software, other studies used more than one expert for these measurements [21,22], and others obtained them through automatic or semi-automatic methods [17,20,44]. The use of a single investigator is indeed one of the limitations of this pilot study since it may introduce some bias in the ChT manual measurements through the DRI-OCT Triton^TM^ OCT software. In future studies, it should be considered to use an average value of each measurement obtained from multiple investigators for more reliable values. However, some studies using the same OCT system and measurement of choroidal thickness have shown excellent inter-session and inter-operator repeatability in healthy subjects.

Most human studies have reported a rapid sub-foveal ChT increase of about 10 and 20 µm [15,16,20,22,34,41,44,45], regardless of the amount of time and myopic defocus the eye was exposed to. In an aged-matched Asian population, Chiang et al. found a significant part of choroidal thickening with +2 D defocus after 10 min, while −2.00 D defocus showed the opposite effect after 20 to 30 min [16]. Some orthokeratology studies showed significant changes in ChT within the first three months of treatment, with no major changes in the following months [21,22,45]. Thus, there appears to be a non-dependence on the defocus’ degree required to induce an increase in ChT, although different types of optical designs may induce different defocus profiles to achieve the effect.

The results of the present study and most others show that the myopic shift induced by positive defocus causes a significant choroidal thickening. Together with the significant reduction in the rate of eye growth observed with myopia management devices, the results help to strengthen the possibility that the choroid may function as a biomarker for ocular growth to signal whether the eye will continue to grow or not through defocus stimulation. Nevertheless, this possibility still needs to be more carefully studied. One aspect that deserves further attention is the connection between the visual signal and the impact on retinal activity that potentially triggers the choroidal response. This pilot study aimed at looking into that domain, and the results are discussed below.

### 4.2. Functional Changes—ERG Findings

Regarding the retinal activity, a decrease in the amplitude of the response of both the outer (b-wave of ffERG photopic 3.0) and inner retina (pattern ERG) was observed with the PG, compared to the SV. Additionally, the PG showed a negative shift of the ffERG photopic 3.0 response.

Although there was a significant decrease in b-wave amplitude, the changes in the ffERG 3.0 photopic response were not very evident. No studies supporting the change in ffERG tests with defocus (or other types of blur) were found in the literature. In a study by Sachidanandam and collaborators [29], the eye length significantly influenced the parameters of the ffERG response, including the photopic 3.0 test, while refractive errors (blur) did not. Since the spaced flashes of light do not contain contrast information, the response from the ffERG stimuli is not sensitive to blur [46].

On the other hand, there was a decrease in both amplitudes of the pattern ERG response with PG. Although no electrophysiological studies used CLs of similar design, these results are consistent with previous findings of a decreasing pattern ERG amplitude with increasing blur level [39,47,48,49,50]. The ganglion cells, whose activity is obtained from pattern ERG records, are sensitive to changes in contrast. Inducing defocus and higher-order aberrations yields decreased contrast, resulting in the attenuation of ganglion cells’ activity as indicated by decreased pattern ERG amplitudes [47,50]. The possible influence of high-order aberrations in the retinal response should also be carefully considered. Although they were not evaluated in this study, the power gradient design induces a change in the magnitude of coma and spherical-like high-order aberrations of the overall image reaching the retina [43]. A study by Panorgias et al. found the spherical aberration to decrease the amplitude of the retinal response than the defocus of the same magnitude, suggesting that the retina might be more sensitive to spherical aberration than defocus. They suggested that this might be due to the difference in the high spatial frequency content between defocus and spherical aberration since spherical aberration could optimize the central retinal response of high spatial resolution content, while defocus does not [51]. Following this, the retinal response changes encountered with the PG might be caused by the combined impact of the induced high-order aberrations and defocus changes in the peripheral retina.

The PG evoked a negative shift of the SV photopic 3.0 ffERG response curve and decreased the b-wave amplitude. Beyond feedback mechanisms, amacrine cells and inter-plexiform cells mediate the response between the inner and outer retina. This, coupled with the fact that ffERG is not affected by low to medium levels of blur, might suggest that the changes observed in pattern ERG response possibly create a retrograde signaling mechanism to bipolar cells. This retrograde signaling phenomenon has been discussed in previous studies and seems to be associated with a specific type of ganglion cells, the intrinsically photosensitive retinal ganglion cells [52,53,54]. A study on human eyes suggested a potential involvement of the retrograde mechanism after observing a simultaneous increase in pattern ERG amplitude and ffERG photopic 3.0 b-wave 20 min after stimulation of the optic nerve head with blue light in myopic eyes [55,56], but not in non-myopic eyes [56]. Nevertheless, those changes were evoked by a spectral selective light stimulus (450 nm wavelength), while in the current study, the ERG changes were induced by a change in the optical quality and contrast of the retinal image.

Overall, the present study shows that retinal electrophysiology is sensitive to the retinal activity changes induced by optically driven myopia control devices and opens a route for developing short-term biomarkers to test the potential impact of such devices in the longer term.

### 4.3. Relationship between Structural and Functional Changes

The relationship between functional and structural retinal changes has been studied and was one of the goals of the present study. However, given the exploratory nature of this study, such potential connections were established with caution. Most studies report very low values of coefficient of determination, which, contrary to what one might expect, suggests that structural choroidal changes are not an exclusive factor of retinal functionality, nor the most relevant. Park et al. [27] observed a decrease in the amplitude of multifocal ERG associated with a thinning of the mid-inner retinal layers with the level of myopia, especially in a particular parafoveal region. However, their results showed that only 20% of the changes in amplitude could be attributed to the differences in those retinal layers’ thickness. The present study seems to be the first one to look simultaneously for possible changes in ChT and in the retinal functionality driven by a device that might have a significant impact on myopia control [57]. The moderate correlations observed in this study showed that as the choroid becomes thicker, the outer retinal activity decrease (decrease in a- and b-wave amplitude). However, the lack of representativeness and the data dispersion might induce false or biased conclusions since such correlations between ERG parameters and retinal layer thickness in healthy myopes were reported to be low [26,27].

Other tests to analyze retinal functional changes could have been used and possibly allowed for deriving more definitive conclusions from this pilot study. Since ganglion cells would be more sensitive to contrast changes, and ffERG could produce clearer results on the photoreceptor and bipolar cells’ activity, the response of the outermost and innermost cells was analyzed separately. However, global-flash mfERG allows to simultaneous extract the response of outer and inner layers since it distinguishes two components of the response: the direct component (DC), with a higher contribution from photoreceptors and bipolar cells, and the induced component (IC), with a higher contribution from amacrine and ganglion cells. Oscillatory potentials are more sensitive to changes between the metabolic and vascular needs of the retina, so a- and b-waves are not normally affected [58]. As such, their recordings could have been a more assertive and feasible option to investigate possible changes in the retinal activity with the increase in ChT observed with the PG. Furthermore, it could be relevant to analyze the relationship between structural and physiological retinal changes with myopia control devices that have proven their effectiveness in children. This could help to unravel the mechanisms of eye growth regulation associated with myopia further.

## 5. Conclusions

The choroidal thickness increased in the presence of positive defocus driven by a power gradient CL developed for myopia management. The influence of the peripheral myopic defocus and the increment in spherical and comatic high-order aberrations on the foveal image evoked a higher increase in the sub-foveal ChT than in the periphery after 30 min of lens wear. The decrease in pattern ERG amplitude with peripheral gradient CLs relative to single-vision CLs might be driven by image quality degradation [42]. This, coupled with the decrease in b-wave amplitude without other relevant ffERG photopic 3.0 changes, suggests that there may be a retrograde signaling mechanism from the ganglion cells to the bipolar cells. This warrants further research to elucidate the retinal mechanisms of optically driven myopia control devices.

## Figures and Tables

**Table 1:** Implicit times and amplitudes of a- and b-waves (median (IQR)) from the ffERG photopic 3.0 response with the SV and PG. The right column shows the median (IQR) difference with the statistical significance (Wilcoxon test).

		SV	PG	diff *p*-Value
Implicit time (ms)	a-wave	17.32 [1.25]	17.47 [1.18]	0.15 [1.20] 0.512
	b-wave	32.29 [1.47]	33.03 [1.32]	0.50 [0.60] **0.049**
Amplitude (µV)	a-wave	38.39 [16.71]	28.20 [10.67]	0.25 [15.75] 0.093
	b-wave	112.41 [63.24]	105.62 [44.85]	11.80 [30.55] **0.047**

SV—single-vision contact lens; PG—myopia control radial power gradient contact lens; diff—difference; ms—milliseconds; µV—microvolts; In bold—*p*-value ≤ 0.050, Wilcoxon test.

## Data Availability

The data presented in this study are available on request from the corresponding author.
